# The Effects of Marine Carbohydrates and Glycosylated Compounds on Human Health

**DOI:** 10.3390/ijms16036018

**Published:** 2015-03-16

**Authors:** Hee-Kyoung Kang, Chang Ho Seo, Yoonkyung Park

**Affiliations:** 1 Department of Biomedical Science, Chosun University, Gwangju 501-759, Korea; E-Mail: hkkang129@gmail.com; 2 Department of Bioinformatics, Kongju National University, Kongju 314-701, Korea; E-Mail: chseo@kongju.ac.kr; 3 Research Center for Proteineous Materials, Chosun University, Gwangju 501-759, Korea

**Keywords:** marine organisms, carbohydrate, glycoside, antioxidant, antibacterial, anticoagulant, antiviral, anti-inflammatory, immune system

## Abstract

Marine organisms have been recognized as a valuable source of bioactive compounds with industrial and nutraceutical potential. Recently, marine-derived carbohydrates, including polysaccharides and low molecular weight glycosylated oligosaccharides, have attracted much attention because of their numerous health benefits. Moreover, several studies have reported that marine carbohydrates exhibit various biological activities, including antioxidant, anti-infection, anticoagulant, anti-inflammatory, and anti-diabetic effects. The present review discusses the potential industrial applications of bioactive marine carbohydrates for health maintenance and disease prevention. Furthermore, the use of marine carbohydrates in food, cosmetics, agriculture, and environmental protection is discussed.

## 1. Introduction

Marine organisms are an important source of bioactive molecules that have been used to treat various diseases. Because of these benefits, much effort has focused on the isolation and characterization of biologically active natural products. The relatively small number of marine organisms studied thus far has yielded thousands of new chemical compounds. Unusual marine environments are associated with chemical diversity, resulting in novel active substances for the development of bioactive products [1,2,3,4].

Oceans cover more than 70% of the earth’s surface, representing an enormous resource for the discovery of potential therapeutic agents. Over the last several decades, numerous compounds with interesting pharmaceutical activities have been identified in marine organisms [1,5,6]. Thus, marine organisms may be a potential source of essential and novel biologically active substances for the development of therapeutics. The diversity of marine environments provides a unique source of bioactive chemical compounds that could lead to potential new drug candidates.

Marine carbohydrates vary enormously in structure; however, they remain under-exploited and require further study to establish their potential use in novel therapeutics [4]. Carbohydrates have a wide variety of biotechnological applications. Nevertheless, preparation of high quality complex carbohydrates, such as those found in marine life, can be challenging. In order to use these carbohydrates in food supplements and cosmetics, among other applications, active research is necessary to overcome the current limitations [7]. Furthermore, marine-derived polysaccharides have a variety of bioactivities, including antitumor, antiviral, anticoagulant, antioxidant, and anti-inflammatory effects.

In this review, we will present the structures and activities of bioactive marine-derived carbohydrates, including polysaccharides and low molecular weight glycosylated oligosaccharides, which have been isolated from sponges, algae, bacteria, and fungi from 2006 to the present.

## 2. Type of Sugar-Bound Compounds from Marine Organisms

### 2.1. Polysaccharides and Oligosaccharides

Polysaccharides represent a structurally diverse class of biological macromolecules composed of monosaccharide (sugar) polymers linked through glycosidic bonds. This structural diversity arises from the different sugars and sugar derivatives, such as uronic acid, found in polysaccharides, and because each sugar can be covalently linked to others at several different positions on the sugar ring. They are used extensively as foods and pharmaceuticals. Furthermore, the enormous variety of polysaccharides extracted from marine plants and animal organisms, or produced by marine bacteria and fungi, has resulted in a constantly evolving group of potential bioactive compounds [8,9].

Many marine organisms produce polysaccharides that have diverse applications, due to their biofunctional properties, and much research has been conducted to assess the possible use of these polysaccharides. Marine polysaccharides, including cellulose, fucan, glucosaminglycan, glucan, chitin, chitosan, laminaran, carrageenan, agar, and alginic acid, have anti-oxidative, antibacterial, antiviral, antitumor, immunostimulatory, and anticoagulant effects. Thus, these compounds could potentially be developed into therapeutics and nutraceuticals [8,10,11,12,13].

Oligosaccharides are low molecular weight carbohydrates found in nature that are larger than simple sugars, but smaller than polysaccharides. The number of sugar moieties in oligosaccharides varies; however, they generally contain 10–12 monosaccharides. Nevertheless, some rhamnogalacturonans, which contain 30 or more monosaccharides, are termed megaoligosaccharides [14]. The smallest structure that retains bioactivity remains unclear, due to unknown specificity; however, a trisaccharide has shown activity in some vaccines [15]. Oligosaccharides can be obtained by direct extraction from natural sources or chemical processing, such as polysaccharide hydrolysis or chemical synthesis from disaccharides.

Marine oligosaccharides are generated by chemical or enzymatic hydrolysis of marine polysaccharides, which are mainly extracted from seaweeds and other sea organisms. Marine polysaccharides are generally classified into three types according to their source: plant polysaccharides [16], animal polysaccharides [17], and microbial polysaccharides [18]. The chemical compositions and structures of most marine oligosaccharides are complex and heterogeneous. A recent review on the biofunctions of marine oligosaccharides has increased interest in their application for novel therapeutics [19,20]. Moreover, these oligosaccharides have many beneficial effects, and thus could be developed into nutraceuticals. Marine oligosaccharides, including chitosan oligosaccharides, and fucoidan oligosaccharides, have been used in a variety of applications, including food production, cosmetics, biomedicine, agriculture, environmental protection, and wastewater management [19,20,21,22].

### 2.2. Glycosides

Many natural products derived from marine organisms have biological activities and low toxicity, rendering them suitable for administration in order to exert a diverse range of effects. Glycosides are compounds which contain a sugar bound to another functional group via a glycosidic bond. Glycosides therefore include two parts, the sugar and the aglycone, and play numerous important roles in living organisms. The aglycone may be a terpene, flavonoid, coumarine or any other natural molecule. Glycosides are chemically diverse. Natural glycosides may contain a single sugar group (monosaccharide) or several sugar groups (oligosaccharide) [23,24].

Triterpene and steroid glycosides are a class of natural products with both terrestrial and marine origins. Terrestrial members of this class, which are isolated from various plants, include toxic, heart-arresting cardenolide glycosides (cardiac glycosides), spirostan and furostan steroid saponins, and pregnane glycosides. Several cardiac glycosides are used therapeutically in the treatment of heart failure and atrial arrhythmia. They also have potential applications in cancer chemotherapy, as evidenced by clinical trials evaluating cardiac glycoside-based anticancer drugs [24]. Many glycoside compounds belonging to other structural groups show cytotoxic, antibacterial, and hypocholesterolemic effects [25,26].

In this review, we have surveyed carbohydrates, including polysaccharides, oligosaccharides, and glycosides from marine sponges, algae, bacteria, fungi, and fish, which have shown efficacy or activity against infectious diseases, including bacterial, fungal, and viral infections, from 2006 to the present.

## 3. Biological Activities of Marine Carbohydrate and Glycosides

Table 1 presents novel bioactive carbohydrates and glycosides reported from 2006 to the present, along with their composition, source of origin, and potential for bioactivity.

### 3.1. Antioxidant Activity

Antioxidants protect organisms against oxidative damage caused by reactive oxygen species (ROS), such as hydrogen peroxide (H_2_O_2_), the superoxide anion (O_2_^−^), and hydroxyl radicals (OH^−^) [27,28]. They may cause significant damage to cell structures, contributing to lipid peroxidation or the formation of DNA adducts that promote cancerous mutations or cell death [29,30]. To reduce damage to the human body and prolong food storage, synthetic antioxidants are used in industrial processing. Antioxidants can minimize oxidative damage by increasing the natural defenses of the cell and by scavenging free radicals [27,31]. Butylated hydroxyanisole (BHA), butylated hydroxytoluene (BHT), propyl gallate (PG), and tertiary butylhydroxyquinone (TBHQ) are authorized synthetic antioxidants used in food [32]. There are some serious issues concerning the toxicity of these compounds. The main problem is related to their metabolism, and possible absorption and accumulation, in body organs and tissues [33]. Thus, the identification of safe, natural antioxidants that can be produced at low cost, is in high demand. Marine resources have attracted attention in the search for natural antioxidants to develop new medicinal and functional food ingredients, due to the numerous antioxidant compounds with potential free radical scavenging activity.

#### 3.1.1. Polysaccharides from *Hyriopsis cumingii* (HCPS-3)

The antioxidant activity of *Hyriopsis cumingii* polysaccharides (HCPS) was evaluated both *in vitro* and *in vivo*. *In vitro* antioxidant assays revealed that HCPS could scavenge hydrogen peroxide, superoxide, and 2,2-diphenyl-1-picrylhydrazyl (DPPH), chelate the ferrous ion, and reduce the ferric ion. HCPS-3 (**1**) exhibited significantly higher antioxidant activity than crude HCPS, HCPS-1, and HCPS-2 [34]. To test the antioxidant activity *in vivo*, varying doses of HCPS-3 were orally administrated over 15 days to a d-galactose-induced aged mouse model. Administration of HCPS-3 significantly inhibited the formation of malondialdehyde (MDH) in the livers and serum of mice, and raised the activity of antioxidant enzymes and total antioxidant capacity in a dose-dependent manner, directly and potently inducing antioxidant activity [34].

#### 3.1.2. Crude Endopolysaccharides from *Cerrena unicolor* (c-EPL)

Crude endopolysaccharides (c-EPL, **2**) were isolated from the idiophasic cultures of the white rot fungus *Cerrena unicolor*. The scavenging ability of the c-EPL was determined to be between 36% and 70% by chemiluminescence, inhibiting 2,2'-azino-*bis*(3-ethylbenzothiazoline-6-sulphonic acid) (ABTS) activity 2%–60% and DPPH activity 28%–32% following treatment with 6.25–800 μg/mL. Preliminary toxicity tests were performed using the Microtox system, revealing that c-EPL inhibits *Vibrio fischeri* (85.37%) and *Staphylococcus aureus* (18.96 μg/disk) growth [35].

#### 3.1.3. Polysaccharides from *Mytilus coruscus* (MP-I)

**Figure 1:**
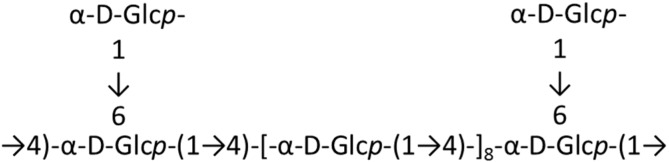
Predicted structure of MP-I (**3**), isolated from *Mytilus coruscus* [36].

Xu *et al.* [36] isolated and characterized the antioxidant polysaccharide MP-I, composed of glucose monomers (**3**), from *Mytilus coruscus*. This polysaccharide was shown to be a branched α-(1→4)-d-glucan, with a single α-d-glucose at the C6 position every eighth residue along the main chain (Figure 1). This molecule (1.35 × 10^6^ Da) protected against acute liver injury in mice. MP-I dose-dependently decreased serum alanine aminotransferase (ALT), serum aspartate aminotransferase (AST), and hepatic MDA levels, increased the total hepatic superoxide dismutase (T-SOD) activity, and improved hepatic damage following CCl_4_-induced liver injury in mice. The observed bioactivity indicates that MP-I is a beneficial marine carbohydrate, which may have pharmaceutical applications in humans [36].

#### 3.1.4. Polysaccharides from *Mytilus edulis* (HWS)

Polysaccharides from *Mytilus edulis* (HWS, **4**) were extracted using water, acidic conditions, and alkaline conditions, with extraction yields of 7.9%, 3.5%, and 8.3%, respectively. HWS is a glucan with an α(1→4)-linked α-d-Glc*p* backbone, with some (1→2,4)-linked-α-d-Glc*p* and →6)-β-Glc-(1→ side chains [37]. On average, there was one branch for every six backbone residues. The antioxidant activity was evaluated *in vitro* by a spectrophotometric assay, revealing that HWS had enhanced hydroxyl radical, superoxide anion radical, and nitrite scavenging effects, as well as reducing capacity. HWS obtained by different extraction methods showed varying antioxidant activity, with water and alkali extracts exerting stronger effects than acid extracts [37].

#### 3.1.5. Polysaccharides from *Penicillium* sp. F23-2 (PS1-1, PS1-2 and PS2-1)

Three polysaccharides, PS1-1 (**5**), PS1-2 (**6**) and PS2-1 (**7**), were isolated from the marine fungus *Penicillium* sp. F23-2, and their antioxidant activity was assayed *in vitro*. PS1-1, PS1-2, and PS2-1 primarily consisted of mannose with variable amounts of glucose and galactose; however, the polysaccharides differed in their glucuronic acid content, molecular weights, and glycosidic linkage patterns [38]. The three polysaccharides possessed good antioxidant effects, with an ability to scavenge superoxide radicals and hydroxyl radicals. Furthermore, the three polysaccharides scavenged DPPH radicals with EC_50_ (the median effective concentration) values in the range of 2.53–6.81 mg/mL. PS2-1 had the highest antioxidant activity, suggesting that a relatively low molecular weight and higher glucuronic acid content may increase antioxidant activity. The differences in the antioxidant abilities of the polysaccharides were directly related to differences in their chemical features [38].

#### 3.1.6. Sulfated Polysaccharide from *Sargassum swartzii*

Vijayabaskar *et al.* described a sulfated polysaccharide (**8**) from the brown algae, *Sargassum swartzii* [39]. This sulfated polysaccharide showed a high percentage of carbohydrate (7.40% ± 0.63%), and sulfate (5.3% ± 1.54%). The highest antioxidant activity was observed in ABTS (55% ± 3.61%), followed by H_2_O_2_ (47.23% ± 2.81%) and DPPH (25.33% ± 2.52%). This sulfated polysaccharide also inhibited both Gram-positive and Gram-negative bacteria (zone of inhibition: 2–16 mm disk) [39]. The molecular weight of the sulfated polysaccharide was found to be 50 kDa. This sulfated polysaccharide from *Sargassum swartzii* could provide a valuable natural antioxidant source, as well as acting as an antibacterial agent.

#### 3.1.7. Chitin Oligosaccharides from Crab Chitin Obtained by Acid Hydrolysis (NA-COS)

Chitin, a long-chain polymer of *N*-acetylglucosamine, is one of the most abundant polysaccharides and is usually prepared from the shells of crabs and shrimps [40]. Chitin oligosaccharides (NA-COS, **9**) with a low molecular weight distribution (229.21–593.12 Da) were produced from crab chitin by acid hydrolysis to improve the water-solubility and biological activity [41]. The IC_50_ values of NA-COS against DPPH, hydroxyl radicals, and alkyl radicals were 0.8, 1.75, and 1.14 mg/mL, respectively [10]. Furthermore, NA-COS inhibited ROS-induced DNA damage in human lymphoma U937 cells and directly scavenged radicals in human fibrosarcoma cells (HT1080), as assessed by 2',7'-dichlorofluorescin diacetate (DCFH-DA). NA-COS has antioxidant effects in live cells, and could potentially be used in food supplements or nutraceuticals [41].

#### 3.1.8. Floridoside and d-Isofloridoside

**Figure 2:**
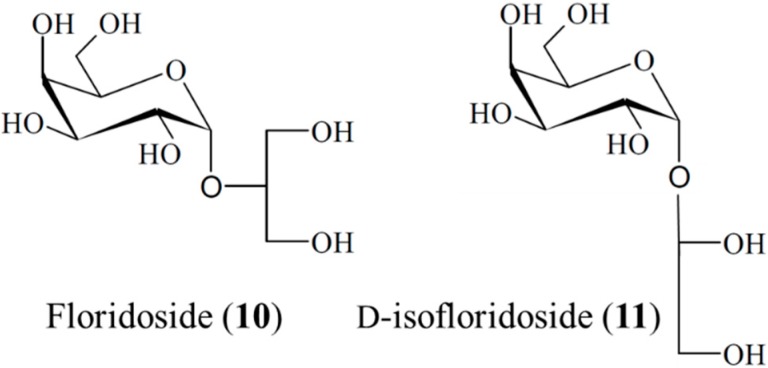
Floridoside (**10**) and d-isofloridoside (**11**), isolated from the marine alga *Laurencia undulata* [42].

Li *et al.* [42] reported that floridoside (**10**) and d-isofloridoside (**11**) isolated from the South Korean marine red alga *Laurencia undulata* possessed significant antioxidant capacity and inhibited the pro-inflammatory matrix metalloproteinases, MMP-2 and MMP-9. These data suggest that floridoside and d-isofloridoside are candidates for further development as natural marine antioxidants [42]. Furthermore, floridoside (**10**) and d-isofloridoside (**11**) could be used in foods and pharmaceuticals as natural marine antioxidants [42]. Furthermore, structure-activity relationships (SARs) could be elucidated based on the structural characteristics of floridoside and d-isofloridoside. These two glycosides are isomers (Figure 2); the galactose group and glycerol residue can donate a hydrogen ion easily, and then excited hydroxyl groups can attract electrons easily. This provides these isolates with more effective antioxidant activity. Meanwhile, small differences can also be found between these compounds due to the different connection positions. Both isomers could contribute to the efficacy of natural marine antioxidants in food and pharmaceutical applications [42].

### 3.2. Antidiabetic Activity

Diabetes mellitus is the most common endocrine disorder, with more than 150 million people suffering worldwide [43]. Literature surveys indicate more than 400 plant species have anti-diabetes activity, and most natural products used for diabetes treatments have been isolated from plants [44]. In contrast, the antidiabetic effects of compounds derived from marine bacteria and fungi remain poorly investigated, but may be of great promise in the search for novel drugs.

#### 3.2.1. Fucoidan

**Figure 3:**
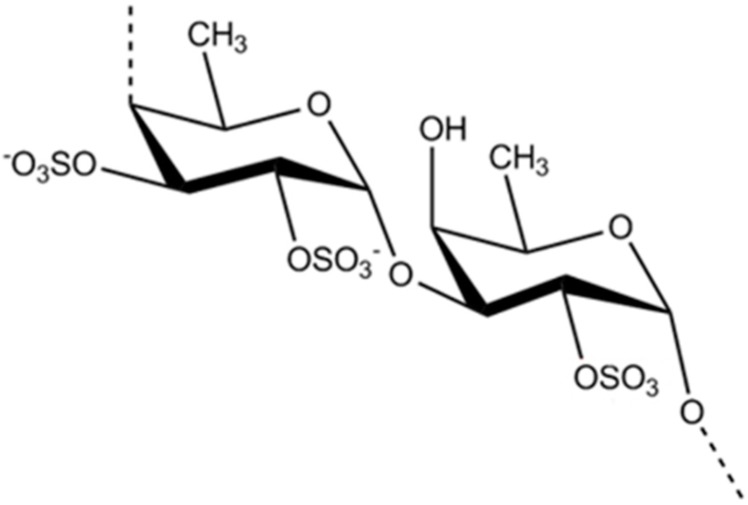
The structure of fucoidan (**12**) [46].

Fucoidan (**12**) is primarily extracted from marine algae such as *Fucus vesiculosus*, *Ecklonia kurome*, and *Undaria pinnatifida*. Fucoidan consists of l-fucose together with xylose, galactose, and mannose [45]. Since the identification of fucoidan, it has been broadly studied for its numerous biological effects. Kim *et al.* [46] studied the effects of different molecular weight forms of fucoidan (Figure 3, 5 kDa, 5–30 kDa, and crude) using an oral glucose tolerance test in mice. Blood glucose levels were strongly suppressed and insulin sensitivity was improved following fucoidan treatment. Furthermore, the results demonstrated that fucoidan activity is dependent on its molecular weight, with low molecular weight fucoidan (LMWF) strongly reducing hyperglycemia in diabetes [46]. Jeong *et al.* also investigated the metabolic effects of LMWF in obese diabetic mice following oral administration for six weeks [47]. By determining the blood glucose levels, total cholesterol levels, fat adiponectin content, and related indicators in blood and tissue samples, LMWF was shown to improve glucose homeostasis and lipid profiles in *db*/*db* mice and endoplasmic reticulum stress-induced insulin resistant L6 myotubes in a manner similar to metformin [47]. Fucoidan extracted from brown seaweeds, which have a higher sulfate content, was reported to have anti-obesity [48] and anti-inflammatory activity in several cell lines [49].

#### 3.2.2. Aquastatin A

**Figure 4:**
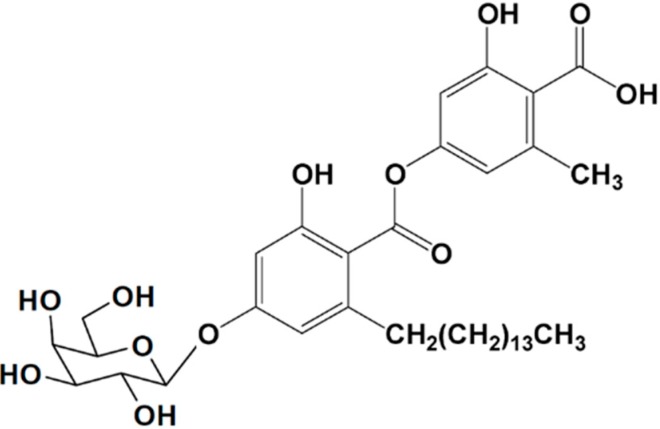
Aquastatin A (**13**), isolated from marine-derived fungus *Cosmospora* sp. SF-5060 [50].

Aquastatin A (**13**), a protein tyrosine phosphatase 1B (PTP1B) inhibitor, was identified in the EtOAc extract of the culture broth of the marine-derived fungus *Cosmospora* sp. SF-5060 (Figure 4) [50]. Aquastatin A potently inhibits PTP1B with an IC_50_ value of 0.19 μM. Protein tyrosine phosphatases (PTPs) constitute a large family of enzymes, which are responsible for the modulation of tyrosine phosphorylation-dependent cellular events. Studies demonstrated that PTP1B, an intracellular non-receptor type PTP, negatively regulates insulin- and leptin-receptor mediated signaling pathways. Thus, its inhibition may represent an outstanding, novel therapy for type 2 diabetes and obesity. Aquastatin A (**13**) also modestly, but selectively inhibited PTP1B over other protein tyrosine phosphatases, such as TCPTP, SHP-2, LAR, and CD45. In addition, hydrolyzing studies of the compound suggested that the dihydroxypentadecyl benzoic acid moiety in the molecule was responsible for its inhibitory activity [50]. Therefore, aquastatin A could be viewed as a potential lead compound for the treatment of diabetes and obesity.

### 3.3. Antibacterial Activity

Antibiotics used in the treatment of infectious diseases provide an immeasurable benefit; however, increasing bacterial resistance to antibiotics is of great concern. The dwindling options for anti-infective treatments have renewed interest in the research and development of novel strategies to prevent infection. The pharmaceutical industry has responded by exploiting natural resources to obtain drugs with expanded antibacterial spectrums over the past decade [19,51]. Some of these marine polysaccharides and glycosides have shown interesting antimicrobial activity.

#### 3.3.1. Polysaccharides from *Sepia pharaonis*

The *in vitro* antimicrobial activity of the EDTA-extracted polysaccharide from the *Sepia pharaonis* cuttlebone (**14**) was evaluated [52]. The minimum inhibitory concentration (MIC) was between 20 and 100 mg/mL. *S. pharaonis* polysaccharides (**14**) inhibited the growth of microorganisms (MICs: 100 mg/mL against *S. aureus* and *Escherichia coli*, 80 mg/mL against *Salmonella typhii*, *Vibrio cholerae*, *Klebsiella oxytoca* and *Salmonella paratyphi*, 60 mg/mL against *Proteus mirabilis*, and 40 mg/mL against *Staphylococcus pyogenes*) [52].

#### 3.3.2. Gladius Polysaccharide

The Gladius polysaccharide (**15**) was obtained from two types of squid, *Loligo duvauceli* and *Doryteuthis sibogae*, from Cuddalore and Mudasalodai on the southeast coast of India [53]*.* The MIC values of the fractionated polysaccharide from *D. sibogae* against bacterial strains, including *Bacillus subtilis*, *Shigella* sp., *S.* *typhii*, *Vibrio* *parahaemolyticus*, *Klebsiella pneumonia*, and *E. coli* were 80, 100, 100, 100, 80, and 80 mg/mL, respectively. Furthermore, polysaccharides from *L. duvauceli* had an MIC of 100 mg/mL against *E. coli*. The MIC values for fungal strains, such as *Aspergillus flavus* and *Rhizopus* sp., were 80 and 100 mg/mL in *L. duvauceli*, and 60 and 100 mg/mL in *D. sibogae*, respectively [53]*.*

#### 3.3.3. Caminosides A–D

**Figure 5:**
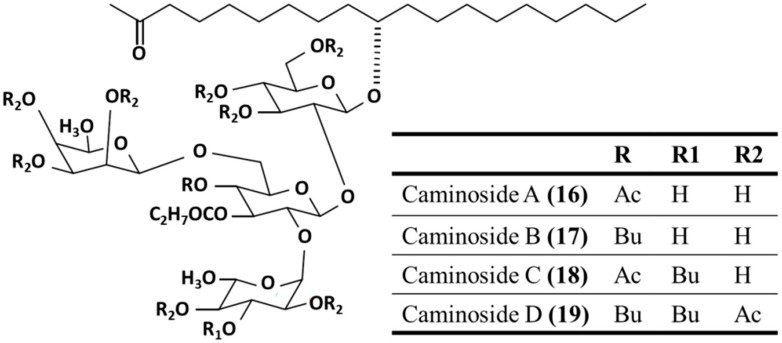
The caminosides A–D (**16**–**19**), isolated from the Caribbean marine sponge *Chaetomorpha sphaeoroconia* [54].

The caminosides A–D (**16**–**19**), isolated from the Caribbean marine sponge *Chaetomorpha sphaeoroconia*, inhibited the pathogenic *E. coli* type III secretion system [54]. This secretory system is found in disease-causing bacteria, but not in their non-pathogenic counterparts. The virulence of pathogens such as the enteropathogenic *E. coli* and enterohemorrhagic *E. coli* 0157:H7, which can be lethal in children and the elderly, is mainly dependent on the *E. coli* type III secretion system [55]. Components of this system may therefore provide effective targets for novel antimicrobial agents; selective type III secretory system inhibitors are potential antimicrobials. Caminosides form inseparable mixtures of bioactive glycolipids that have the same tetrasaccharide chain, but differ in acylation levels. Their carbohydrate chains consist of two d-glucopyranose, one l-quinovopyranose and one 6-deoxy-d-talopyranose carbohydrate units. The middle glucose residue is fully substituted. Interestingly, 6-deoxytalose and l-quinovose are rare in nature. Furthermore, the monosaccharide units are partly acylated (Figure 5) [54,56]. Caminosides A (**16**), B (**17**) and D (**19**) are effective against Gram-positive methicillin-resistant *S. aureus* (MIC = 12.5, 6.3, and 6.3 μg/disk, respectively) and vancomycin-resistant *Enteroccocus* (MIC = 6.3, 3.1, and 6.3 μg/disk, respectively), but show no activity against the Gram-negative bacterium *E. coli* [54].

#### 3.3.4. Sulfate Galactan from *Caminus aerea*

The *in vitro* antimicrobial activity of the marine green algae *Chaetomorpha aerea* has been investigated against Gram-positive bacteria, Gram-negative bacteria, and a fungus [57]. A water-soluble extract of this organism was composed of a sulfated (6.3%) galactan (**20**) with a molecular weight of 1.160 × 10^6^ Da. The polysaccharide was composed of 18% arabinose, 24% glucose, and 58% galactose. The sulfated galactan exhibited selective antibacterial activities against *B. subtilis*, *Micrococcus lutens*, and *S. aureus* (diameters of zones of inhibition: 11–14 mm). The MIC and minimum bactericidal concentration (MBC) showed that this sulfated galactan could be a useful bactericidal agent against *S. aureus* (40 mg/mL) [57].

### 3.4. Antifungal Activity

Resistance to anti-fungal drugs is increasing; thus, novel therapeutic strategies are urgently needed. Structurally unique carbohydrates that are specific to non-mammalian organisms are crucial to the survival of many pathogens. Indeed, inhibiting or interfering with the correct biosynthesis of oligosaccharide materials could be an attractive and highly selective strategy for the development of new anti-fungal agents [58,59]. This suggests that, in principle, new antifungal agents with carbohydrate structures are in development. Since 2006, 16 studies reported antifungal marine carbohydrate-based structure isolated from a diverse group of organisms, which have contributed to the global health challenge posed by drug-resistant fungi.

#### 3.4.1. Novel Triterpene Glycoside, Holothurin B and Holothurin A

**Figure 6:**
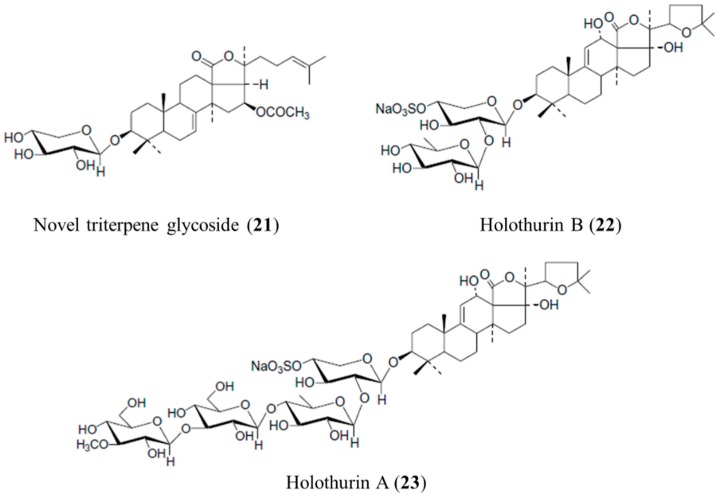
The novel triterpene glycoside (**21**), holothurin B (**22**) and holothurin A (**23**) isolated from sea cucumber *Actinopyga lecanora* [60].

Bioassay-guided fractionation of the sea cucumber *Actinopyga lecanora* methanol extracts led to the isolation of a novel triterpene glycoside (**21**), holothurin B (**22**), and holothurin A (**23**) [60]. The structure of the novel triterpene glycoside (**21**) was shown to be 3-*O*-β-d-xylopyranosyl-16β-acetoxyholost-7-ene (Figure 6). This naturally occurring holothurin contains one sugar unit. Novel triterpene glycoside (**21**) and holothurin A (**23**) showed significant antifungal activity against *Candida albicans* (MIC = 50 μg/mL), *Cryptococcus neoformans* (MIC = 25 μg/mL), *Sporothrix schenckii* (MIC = 25 μg/mL), *Trychophyton mentagrophytes* (MIC = 25 μg/mL), and *A. fumigatus* (MIC = 25 μg/mL) [60].

Comparing the antifungal activity of holothurin B (**22**) with fluconazole, it was observed that holothurin B inhibits *T. mentagrophytes* more potently than fluconazole (MIC = 1.56 μg/mL). In contrast, the MICs of holothurin B and fluconazole were comparable against *S. schenckii* (MIC = 1.56 μg/mL) and *A. fumigatus* (MIC = 3.12 μg/mL) [60]. Marine natural products isolated from the sea cucumber *(Actinopyga lecanora*) continue to be an interesting source of antifungal compounds. Holothurin B could be a lead molecule in the development of a potent drug for the treatment of fungal infections.

#### 3.4.2. Marmoratoside A, 17α-Hydroxy Impatienside A, Marmoratoside B and 25-Acetoxy Bivittoside D

**Figure 7:**
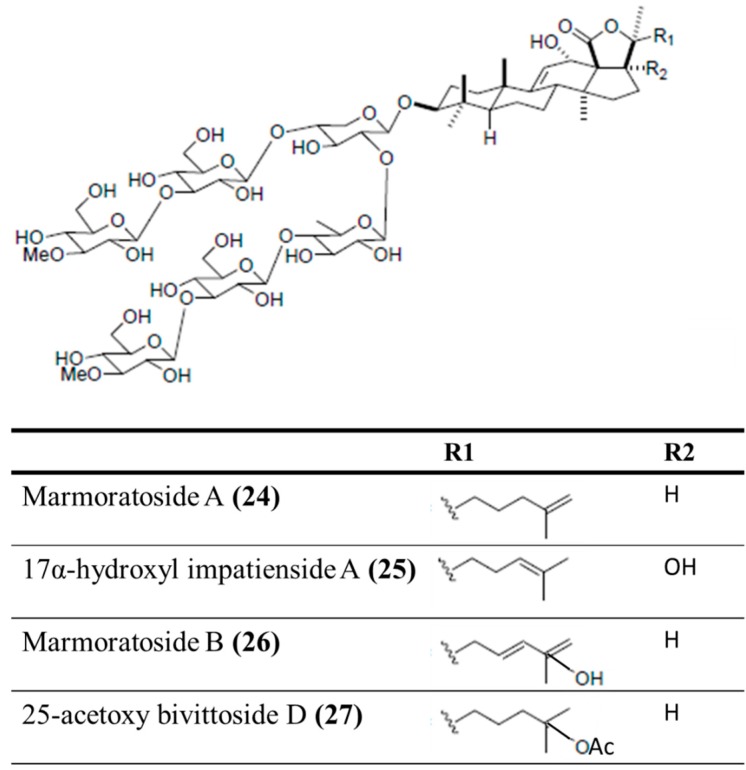
Marmoratoside A (**24**), 17α-hydroxy impatienside A (**25**), marmoratoside B (**26**), and 25-acetoxy bivittoside D (**27**), isolated from sea cucumber *Bohadschia marmorata* [61].

Four holostan-type triterpenoid glycosides, marmoratoside A (**24**), 17α-hydroxy impatienside A (**25**), marmoratoside B (**26**), and 25-acetoxy bivittoside D (**27**), were isolated from the Chinese sea cucumber *Bohadschia marmorata* (Figure 7) [61]. These triterpenoid glycosides exhibited significant activity against six fungal strains: *C. albicans*, *C. neoformans*, *A. fumigatus*, *Trichophyton rubrum*, *Candida tropicalis*, and *Candida krusei* (0.70 ≤ MIC_80_ ≤ 2.81 μM) [61]. The emergence of bacterial resistance to commonly used synthetic antimicrobial (antibacterial and antifungal) drugs, as a result of long-term drug therapy, is increasingly common. Studies of the antimicrobial potential of sea cucumbers have indicated that it would be interesting to explore this natural source of novel antimicrobial agents for the development of drugs to treat infectious diseases.

#### 3.4.3. Scabraside A, Echinoidea A, and Holothurin A1

**Figure 8:**
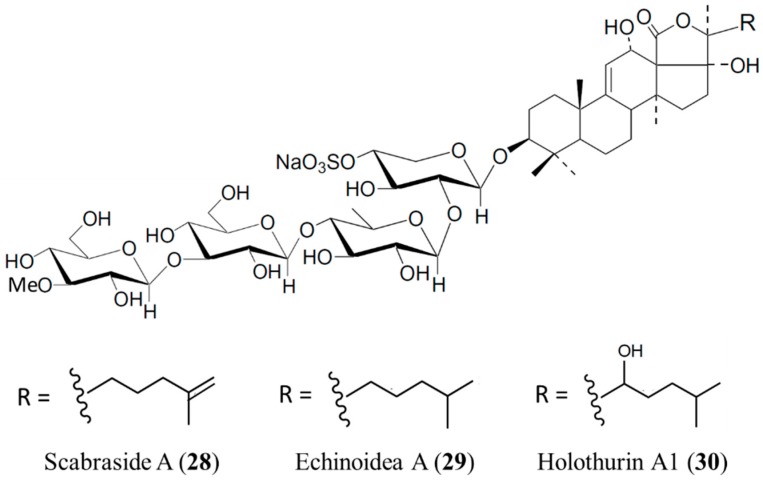
Scabraside A (**28**), echinoidea A (**29**), and holothurin A1 (**30**), isolated from *Holothuria scabra* [62].

Three antifungal triterpene glycosides, scabraside A (**28**), echinoidea A (**29**), and holothurin A1 (**30**), were isolated from *Holothuria scabra* (Figure 8) [62]. These triterpenoid glycosides exhibited significant antifungal activity against seven strains: *C. albicans*, *C. neoformans*, *Candida pseudotropicalis*, *T. rubrum*, *Fonsecaea compacta*, *A. fumigatus*, and *Microsporum gypseum* (1 ≤ MIC_80_ ≤ 16 μg/mL) [62]. The antifungal activity of sea cucumber glycosides is sensitive to their precise functionalization, and sensitivities may vary against different fungi strains. In addition, these glycosides exhibited high cytotoxic activities against human hepatoma (HepG2), human cervical cancer (HeLa), and human leukemia (K562) cell lines, with IС_50_ values of 1–4 μg/mL [63]. However, there were still structure-dependent differences in the cytotoxic activities of different compounds, especially against K562 cells [63].

#### 3.4.4. Sokodoside A and B

**Figure 9:**
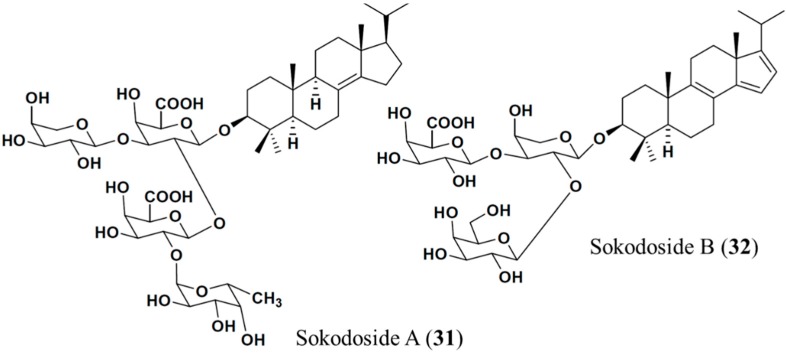
Sokodosides A (**31**) and B (**32**) isolated from marine sponge *Erylus placenta* [64].

Sokodoside B (**32**), isolated from the marine sponge *Erylus placenta*, is a branched trioside with a carbohydrate chain consisting of α-l-arabinose, β-d-galactose, and β-d-galactouronic acid (Figure 9). In the original paper, the configuration of the arabinose residues was erroneously described as β, whereas the coupling constant in the ^1^H-NMR spectra of Sokodoside A (**31**) and B (**32**) revealed an α-configuration of the l-arabinose residues [64]. Sokodoside A (**31**) and B (**32**) show moderate growth-inhibitory activity against the fungus *Mortierella ramanniana* and different strains of the yeast *Saccharomyces cerevisiae*, with or without mutations (cdc28, act1-1 and erg6). Sokodoside B was more active than Sokodoside A. Furthermore, sokodosides A and B show moderate cytotoxicity against P388 cells, with IC_50_ values of 50 and 100 μg/mL, respectively. Hence, there is a correlation between their antifungal and cytotoxic activities [64].

#### 3.4.5. Variegatusides C–F

Four triterpene glycosides, variegatusides C–F (**33**–**36**), were isolated from the sea cucumber *Stichopus variegates* Semper (Holothuriidae), collected from the South China Sea [65]. Variegatusides C–F exhibit the same structural feature, a 23-hydroxyl group at the holostane-type triterpene aglycone side chain. Variegatuside C (**33**) is 3β-*O*-[3-*O*-methyl-β-d-glucopyranosyl-(1→3)-β-d-xylopyranosyl-(1→3)-β-d-glucopyranosyl-(1→2)-β-d-xylopyranosyl]-23(*S*)-hydroxylholo sta-7,24-diene. The structure of variegatuside D (**34**) was deduced as 3β-*O*-[3-*O*-methyl-β-d-glucopyranosyl-(1→3)-β-d-xylopyranosyl-(1→4)-β-d-glucopyranosyl-(1→2)[β-d-glucopyranosyl-(1→4)]-β-d-xylopyranosyl]-23(*S*)-hydroxylholosta-8(9)-ene. Variegatuside E (**35**) was identified as 3β-*O*-[3-*O*-methyl-β-d-glucopyranosyl-(1→3)-β-d-xylopyranosyl-(1→4)-β-d-glucopyranosyl-(1→2)-[3-*O*-methyl-β-d-glucopyranosyl-(1→3)-β-d-glucopyranosyl-(1→4)]-β-d-xylopyranosyl]-23(*S*)-hydroxylholosta-9(11)-ene. Variegatuside F (**36**) is a native compound from *S. variegates* Semper, which has been reported as desacetylstichloroside B1. These glycosides have no sulfate group in their sugar chain and show potent antifungal activities in *in vitro* assays. These triterpenoid glycosides exhibited significant antifungal activity against six strains: *C. albicans*, *C. neoformans*, *C. pseudotropicalis*, *C. parapsilosis*, *C. tropicalis*, and *Microsporum gypseum* (3.4 ≤ MIC_80_ ≤ 12.5 μg/mL) [65].

### 3.5. Anticoagulant Activity

Disorders in blood coagulation increase the risk of hemorrhage or thrombosis [66]. Anticoagulants prevent coagulation [67], and can be used *in vivo* to treat thrombotic disorders. Heparin, an anticoagulant used widely in venous thromboembolic disorders, was discovered more than fifty years ago. However, heparin has several side effects, including thrombocytopenia, hemorrhage, and ineffectiveness due to congenital or acquired anti-thrombin deficiencies, and inability to inhibit thrombin bound to fibrin [68]. Moreover, heparin is primarily extracted from pig intestines or bovine lungs, where it exists in low levels. Therefore, alternative anticoagulants are necessary for safer and more effective therapy.

#### 3.5.1. Fucoidans

Fucoidans (**12**) are a group of sulfated hetero-polysaccharides found in the cell wall of some members of Phaeophyceae. Drozd *et al.* [69] elucidated the pharmacology of the fucoidans (**12**) from the marine algae *Fucus evanescens* and *Laminaria cichorioides*, showing that these sulfated polysaccharides inhibited both thrombin and factor Xa with a comparable potency to non-fractioned and low-molecular weight heparin. In rats, intravenous injection of fucoidans dose-dependently increased anticoagulant activity in the plasma. Interestingly, fucoidans can form complexes with protamine sulfate. The observed quantitative difference in fucoidan action may result from variations in the degree of sulfation and the presence of different glycoside bonds in the polysaccharide molecules.

Shanthi *et al.* extracted two fucoidan fractions from the brown seaweed *Turbinaria decurrens*, found along the coast of Pamban in the Gulf of Mannar, India [70]. Both the low molecular weight (LMW; <3500 Da) and high molecular weight (HMW; >3500 Da) fucoidans showed promising anticoagulation activity using an *in vitro* activated partial thrombin time (APTT) assay. Fucoidan was characterized as containing primarily galactose, followed by fucose, mannose and glucose. However, higher anticoagulation activity was recorded using the HMW fraction than the LMW fraction. Furthermore, the anticoagulation activity was increased with increased fucoidan sulfate content [70].

#### 3.5.2. Acidic Polysaccharide from *Laminaria cichorioides*

The acidic polysaccharide (**37**) derived from the brown algae *Laminaria cichorioides* is a complex and heterogeneous sulfated fucan [71]. Its structure contains a 2,3-disulfated, 4-linked α-fucose unit. The acidic polysaccharide (**37**) potently inhibits coagulation, as shown in an APTT assay, which is mainly mediated by thrombin inhibition through heparin cofactor II. It also accelerates thrombin and factor Xa inhibition by antithrombin, but at a lower potency. Sulfated fucans from *L. cichorioides* are a promising anticoagulant polysaccharide, and could represent an alternative antithrombotic compound, due to its preferential heparin cofactor II-dependent activity [71].

#### 3.5.3. Sulfated Polysaccharides from Brown Algae

SPs with anticoagulant activity were purified from the brown algae *Sargassum tenerrimum* (St, **38**), *Sargassum wightii* (Sw, **39**), *Turbinaria conoides* (Tc, **40**), *Turbinaria ornata* (To, **41**), and *Padina tetrastromatica* (Pt, **42**) in the Bonnemaisoniaceae family, from Mandapam Island, India [72]. The high APTT values indicated that StSP (**38**) had high anticoagulant activity (134 ± 1.73), followed by SwSP (**39**, 122 ± 1) > ToSP (**40**, 117.6 ± 1.52) > TcSP (**41**, 108 ± 1.41) > PtSP (**42**, 89.3 ± 1.52). StSP showed maximum heparinoid activity (25.47 heparin USP units/mg), followed by SwSP (22.52 heparin USP units/mg), ToSP (21.45 heparin USP units/mg), TcSP (19.1 heparin USP units/mg), and PtSP (14.51 heparin USP units/mg). These SPs did not prolong prothrombin time. These findings suggested that SPs inhibited molecular targets within the intrinsic and/or common coagulation pathways, but did not act on the extrinsic coagulation pathway [72].

#### 3.5.4. Fucosylated Polysaccharide Sulfate (AMP-2)

A fucosylated polysaccharide sulfate, AMP-2 (**43**), was isolated from the sea cucumber, *Acaudina molpadioidea*, in southeastern China [73]. AMP-2 was a homogeneous carbohydrate with a relative molecular weight of *ca.* 2.4 × 10^4^ Da; monosaccharide composition analysis indicated that this polysaccharide was composed of 1-substituted-Gal*p*, 1,4-disubstituted-GalN*p* (with the NH_2_ group attached to the 2 position of the glycosyl residue), 1,2-disubstituted-FucS*p* (with the SO_4_^2−^ group attached to the 2-position of the glycosyl residue), and 1,4,6-trisubstituted-Glc*p*, in a molar ratio of *ca.* 0.5:2.0:1.0:3.0, together with a small amount of differently substituted Man*p* [73]. AMP-2 (**43**) increased the anticoagulant activity detected by APTT (34.3 at 0.5 mg/mL), PT (11.8 at 0.5 mg/mL), and TT (18.7 at 0.5 mg/mL) assays [73].

#### 3.5.5. Anticoagulant Polysaccharide from *Lomentaria catenata*

An anticoagulant polysaccharide (**44**) was purified from the red algae *Lomentaria catenata* with a molecular weight ranging from 100 to 500 kDa [74]. The purified anticoagulant polysaccharide (**44**) was primarily composed of galactose, with small amounts of glucose, and was highly sulfated. The anticoagulant activity was assayed using the APTT, prothrombin time (PT), and thrombin time (TT) assays. The APT time was greater than 1000 s at 40 μg/mL, and was more potent than heparin (APTT > 1000 s at 62.5 μg/mL). The anticoagulant compound resulted in prolonged APTT and TT, but had little effect on PT. The results showed that the isolated compound might act on the intrinsic and/or common pathways of the blood coagulation system [74].

#### 3.5.6. *Halymenia floresia* Sulfated Polysaccharides (Hf-SP)

Sulfated polysaccharides (SP) in the red seaweed *Halymenia floresia* (Hf) are heparinoids (**45**) [75]. Hf-SP (**45**) obtained from the aqueous extract of *H. floresia* was evaluated for its anticoagulant activity. Hf-SP3 had the highest sulfate content (37.45%). The anticoagulant activity was evaluated by the APTT using rabbit plasma and expressed in international units per mg of Hf-SP. Hf-SP3 had the highest anticoagulant activity (10.72 IU/mg), suggesting that the sulfate is important for anticoagulation [75].

#### 3.5.7. *Asparagopsis taxiformis* Sulfated Polysaccharide (AtSP)

An SP with anticoagulant activity was purified from the red algae *Asparagopsis taxiformis* (At) in the Bonnemaisoniaceae family [76]. The molecular mass of the purified polysaccharide ranged from 60–500 kDa. The anticoagulant activity of AtSP (**46**) was assayed using APTT and PT assays. AtSP (**46**) had a relative clotting factor of 28.57 (>1000 s ± 3.6) at 48 μg/mL, which was comparable with heparin (60 μg/mL). AtSP (**46**) may be capable of inhibiting both intrinsic and extrinsic blood coagulation pathways. The relative clotting factor assayed by PT was within the range of oral anticoagulant agents [76]. Therefore, AtSP could be developed as a potential oral anticoagulant agent.

#### 3.5.8. *Caulerpa cupressoides* var. *lycopodium* Sulfated Polysaccharides (CuSP)

*Caulerpa cupressoides* var. *lycopodium* is a marine green algae containing three sulfated polysaccharide (CuSP) fractions (SP1, SP2, and SP3) [77]. CuSP2 (**47**) had anticoagulant activity *in vitro* and anti- and pro-thrombotic activity *in vivo*. The average molecular weight of CuSP2 ranged from ~8 to >100 kDa. The effect of CuSP2 (**47**) on coagulation proteases (thrombin and factor Xa) was evaluated in the presence of anti-thrombin (AT) and heparin cofactor II (HCII) using human plasma. CuSP2 interfered with the coagulation system by inhibiting the thrombin activity mediated by AT (IC_50_ = 18 μg/mL), as compared with an IC_50_ of 1 μg/mL for inactivation by unfractionated heparin. CuSP2 also inhibited factor Xa activation by AT. The inhibitory effect of CuSP2 on factor Xa, in the presence of AT, showed an IC_50_ of 24 μg/mL. The same property was also noted for heparin (IC_50_ = 1.7 μg/mL). Both thrombin and factor Xa target proteases were inhibited; however, this effect required a concentration approximately 2.5-fold higher of HCII than thrombin inactivation by AT [77].

### 3.6. Antiprotozoal Activity

Eight marine glycosides were reported to possess antimicrobial activity against protozoa, thus contributing to the ongoing global search for novel agents to treat neglected diseases, including Leishmaniasis, caused by several species of the genus *Leishmania*, and African sleeping sickness, caused by *Trypanosoma brucei rhodesiense*.

#### 3.6.1. Pandaroside G and Pandaroside G Methyl Ester

**Figure 10:**
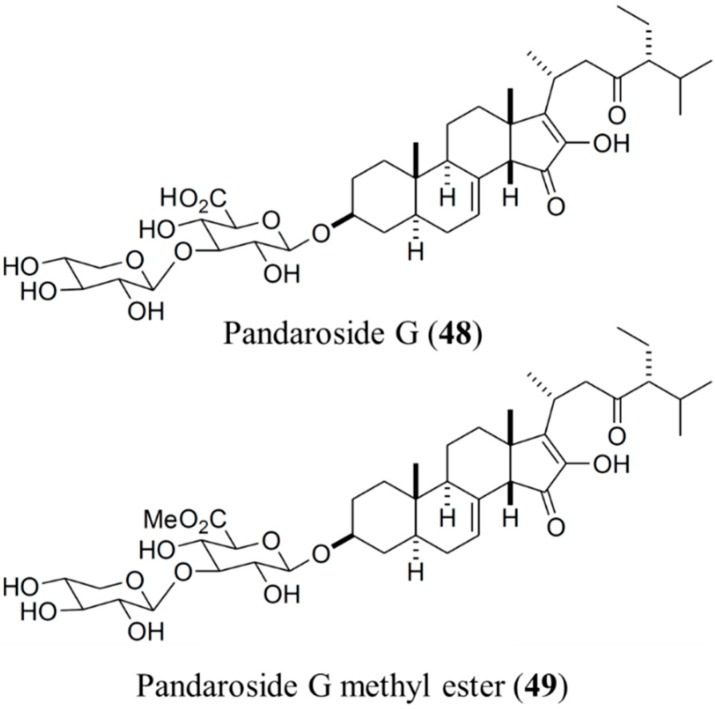
Pandaroside G (**48**) and pandaroside G methyl ester (**49**), isolated from the sponge, *Pandaros acanthifolium* [78].

Regalado *et al.* [78] investigated the Caribbean sponge *Pandaros acanthifolium,* and discovered that, among several novel steroidal glycosides, both pandaroside G (**48**) and pandaroside G methyl ester (Figure 10, **49**) potently inhibited the growth of *T. brucei* *rhodesiense* (IC_50_ = 0.78 and 0.038 μM, respectively), and *Leishmania donovani* (IC_50_ = 1.3 and 0.051 μM, respectively). However, these compounds exhibited high cytotoxicity. In addition, pandaroside G and pandaroside G methyl ester exhibited antimalarial activity against the multidrug-resistant strain of *P. falciparum* (IC_50_ = 2.5 and 13 μM, respectively). When tested in L6 cells, a primary cell line derived from mammalian (rat) skeletal myoblasts, pandaroside G appeared to have cytotoxic potential [78].

#### 3.6.2. Acanthifoliosides A–F

**Figure 11:**
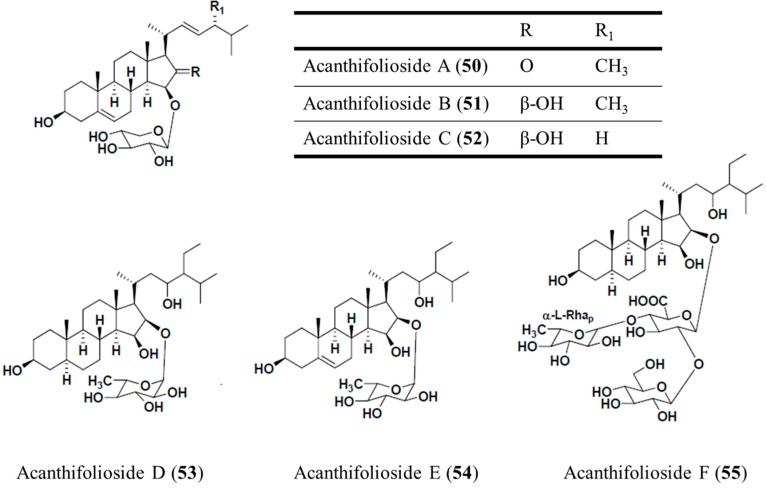
Acanthifoliosides A–F (**50**–**52**) isolated from the sponge, *Pandaros acanthifolium* [79].

Acanthifoliosides A–F (**50**–**55**) were also isolated from the Caribbean sponge *Pandaros acanthifolium* [79]. Acanthifoliosides are structurally related to pandarosides steroid saponins, but they contain common steroid nuclei with trans-junctions between rings C and D. Aglycones of acanthifoliosides A–F were oxidized in ring D. The carbohydrate chains of the glycosides are linked to C-15 in acanthifoliosides A–C or C-16 of the aglycone moieties in acanthifoliosides D–F. Acanthifoliosides A–C (**50**–**52**) are mono-β-d-xylopyranosides, whereas acanthifoliosides D (**53**) and E (**54**) are mono-α-l-rhamnopyranosides. Furthermore, acanthifolioside F (**55**) contains a branched trioside carbohydrate chain (Figure 11). Acanthifoliosides exhibited moderate antiprotozoal activity, inhibiting the growth of *T. brucei* *rhodesiense* (IC_50_ = 6.4–94.8 μM), and *L. donovani* (IC_50_ = 5.7–29 μM) [79].

### 3.7. Anti-Malarial Activity

Malaria is caused by protozoan parasites, most commonly *Plasmodium falciparum*, which are transmitted by the bite of infected mosquitoes. Malaria causes recurrent chills and fever, and kills an estimated 1 million people each year worldwide. Furthermore, it is the most threatening parasitic infection in humans [80,81]. Nevertheless, resistance to commonly used anti-malarial therapeutics is growing. Thus, there is an urgent need to develop new drugs targeting this disease. Moreover, combination treatments should be explored to reduce the risk of resistance. Contributing to the global search for novel antimalarial peptides, and as presented in Table 1, Fourteen novel marine glycosides were shown to possess antimalarial activity since 2006.

#### Glycosides From *Muricea austera*

**Figure 12:**
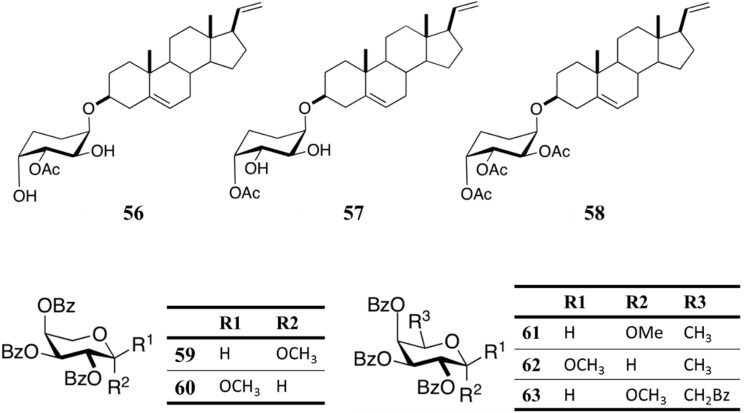
Glycosides **56**–**63**, isolated from octocoral, *Muricea austere* [82].

Bioassay-guided fractionation of the octocoral *Muricea austere* MeOH extract led to the isolation of steroidal pregnane glycosides (**56**, **57**). The structures of glycosides **56** and **57** were identified as 3'-*O*-acetyl-3β-pregna-5,20-dienyl-β-d-arabinopyranoside and 4'-*O*-acetyl-3β-pregna-5,20-dienyl-β-d-arabinopyranoside, respectively (Figure 12) [82]. Glycosides **56** and **57**, showed moderate anti-plasmodial activity (IC_50_ = 67 and 80 μM), which could be somewhat increased by their triacetylated derivative **58** (IC_50_ = 28 μM) (Figure 12). The same authors synthesized and evaluated the anti-plasmodial activity of a series of arabinopyranosides, where two compounds, **59** and **60** (IC_50_ = 35 and 21 μM), were found to be active against *P. falciparum* (Figure 12) [82]. Furthermore, these compounds were more active than the natural arabinopyranosides **56** and **60**. Antiplasmodial activity was also reported in simple sugar derivatives with the same configuration as d-arabinopyranose, including d-fucosides **61** and **62** (IC_50_ = 43 and 36 μM) and d-galactoside **63** (IC_50_ = 29 μM) (Figure 12).

### 3.8. Anti-Viral Activity

A number of infectious diseases caused by viruses have emerged and re-emerged in recent years. Although several antiviral drugs have been specifically developed, drug-resistant mutations are constantly occurring, thus requiring novel antiviral therapeutics. Microalgae have received much attention as potential suppliers of antiviral agents [83,84]. As shown in Table 1, several carbohydrates have been published since 2006 with activity against human cytomegalovirus, the herpes simplex virus (HSV), and the human immunodeficiency virus type-1 (HIV-1), which causes acquired immunodeficiency disease syndrome (AIDS).

#### 3.8.1. Naviculan

*Navicula directa* is a diatom often collected at a deep-sea water sluice gate in Toyama Bay, Japan. Naviculan (**64**), a sulfated polysaccharide isolated from *N. directa* as a novel antiviral agent, consists of fucose, xylose, galactose, mannose, rhamnose, and other trace sugar moieties [85]. Naviculan (**64**) showed inhibited HSV-1 and HSV-2 (IC_50_ = 7–14 μg/mL) by interfering with early stages of viral replication, and inhibiting viral binding, adsorption, and penetration into host cells. Naviculan also showed an inhibitory effect on cell-cell fusion between CD4-expressing and human immunodeficiency virus (HIV) gp160-expressing HeLa cells, which were used as a model of HIV infection [85].

#### 3.8.2. PSC and PBT

Water-soluble sulfated polysaccharides isolated from two red algae, *Sphaerococcus coronopifolius* (PSC, **65**) and *Boergeseniella thuyoides* (PBT, **66**), inhibited the replication of HIV at 12.5 μg/mL *in vitro* by directly controlling the appearance of new viral generations and exerting a virucidal effect [86]. PSC (**65**) and PBT (**66**) are composed of galactose, 3,6-anhydrogalactose, uronic acid, and sulfate in ratios of 33.1%, 11.0%, 7.7% and 24.0% (*w*/*w*) and 25.4%, 16.0%, 3.2%, 7.6% (*w*/*w*), respectively. PSC and PBT were capable of inhibiting the *in vitro* replication of HSV-1 in Vero cells (EC_50_ = 4.1 and 17.2 μg/mL, respectively). These polysaccharides appear to target HSV adsorption to the host cell [86].

#### 3.8.3. GFP and GLPE

The sulfated galactans GFP (**67**), extracted from the red algae *Grateloupia filicina*, and GLPE (**68**), obtained from *Grateloupia longifolia*, also showed antiretroviral activity *in vitro* [87]. GFP and GLPE (the 1,4-α-d-glucan-glucanohydrolase digest of GLP) contained 25.7% and 18.5% sulfate, respectively. The sulfate ester groups were located at carbon 2 for GFP (**67**) and carbons 2 and 6 for GLPE (**68**). Antiretroviral activity was investigated in a primary isolate (PI) of HIV-1 and human peripheral blood mononuclear cells (PBMCs), rather than in T-cell line adapted (TCLA) HIV-1 and T-cell lines, because the former system is more representative of the *in vivo* situation. GFP and GLPE showed potent anti-HIV-1 activity when applied at the time of infection and 2 h post-infection (EC_50_ values = 0.010–0.003 μM; EC_90_ values = 0.87–0.33 μM, respectively), with low cytotoxicity [87]. It is likely that this post-infection activity involved inhibition of early post-absorption steps, such as virus internalization and/or inhibition of cell-to-cell transmission in successive cycles of replication. This finding, together with the observed low cytotoxicity and high selectivity indices in PBMCs, is of relevance to the potential use of these natural compounds in vaginal virucidal formulations.

**Figure 13:**
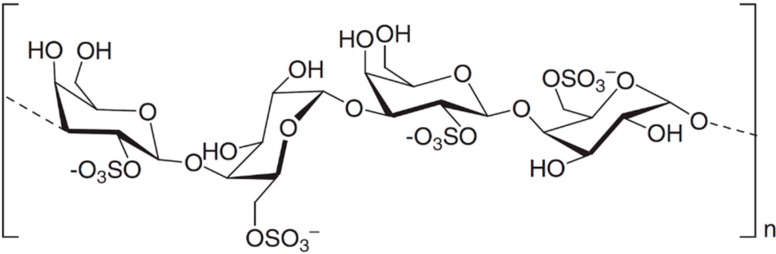
The d,l-galactan hybrid C2S-3 (**69**), isolated from the red seaweed *Cryptonemia crenulata* [88].

#### 3.8.4. d,l-Galactan Hybrid C2S-3

The d,l-galactan hybrid C2S-3 (**69**), isolated from the red seaweed *Cryptonemia crenulata*, is a potent and selective inhibitor of dengue virus (DENV-2) strain multiplication in Vero cells (IC_50_ = 0.8–16 μg/mL), with higher efficacy than heparin [89]. C2S-3 presents the diads [→3]-β-d-(^4^C_1_)-Gal*p*-2-OSO_3_-(1→4)-α-l-(^4^C_1_)-Gal*p*-6'-OSO_3_-(1→] and [→3]-β-d-(^4^C_1_)-Gal*p*-2-OSO_3_-(1→4)-α-d-(^4^C_1_)-Gal*p*-6'-OSO_3_-(1→] (Figure 13). Both disaccharide units present in *C. crenulata* polysaccharides are similar to the repeating structures of the minimal heparin sulfate sequence necessary to interact with glycoprotein C of HSV [87]. The presence of the compound during viral adsorption or internalization exerted a significant and dose-dependent inhibition of DENV-2 plaque number. When the entry of DENV-2 particles into the cell is bypassed, as occurs in virus RNA transfection, the d,l-galactan hybrid C2S-3 (**69**) failed to block the multiplication cycle [89].

#### 3.8.5. Sulfated Xylomannan from *Scinaia hatei*

Mandal *et al.* described a sulfated xylomannan (**70**) isolated from the Indian red seaweed *Scinaia hatei*, which inhibited HSV-1 and HSV-2 (IC_50_ = 0.5–1.4 μg/mL), with low cytotoxicity (CC_50_ ≥ 1000 μg/mL) [89]. Sulfated xylomannan showed very low anticoagulant activity. Furthermore, it had a weak inactivating effect on virions in a virucidal assay at concentrations of 60–100 μg/mL. This compound likely interferes with the HSV-1 multiplication cycle. Sulfated xylomannan (**70**) had 0.4 sulfate groups per monomer unit, an apparent molecular mass of 160 kDa, and contained a backbone of α-(1→3)-linked d-mannopyranosyl residues substituted at C-6, C-4, and C-2 with a single β-d-xylopyranosyl residue. Sulfate groups, when present, are located at C-4 of α-(1→3)-linked d-mannopyranosyl units, and appeared to be very important for the anti-HSV activity of this polymer [89].

#### 3.8.6. Galactofucan Fraction EA1-20

The galactofucan fraction EA1-20 (**71**) from the brown algae *Adenocystis utricularis* exhibited anti-HIV-1 activity *in vitro*, with IC_50_ values of 0.6 μg/mL [90] and low cytotoxicity. The inhibitor effect of EA1-20 (**71**) was not due to an inactivating effect on the viral particle (*i.e.*, no virucidal activity was detected) but rather to a blockade of early events in viral replication. Given these encouraging results, these seaweed-derived fractions provide good candidates for further studies on their potential utility for *in vivo* therapy and/or HIV-1 prophylaxis. EA1-20 has shown potent anti-HSV-1 and HSV-2 activity, in the absence of anticoagulant or antibacterial properties [90]. The latest observations stress this specificity for antiviral activity, reducing the risk of side effects associated with the use of these compounds [89].

#### 3.8.7. Sulfated Fucans

Sulfated fucans (**72**) from the seaweed species *Dictyota mertensii*, *Lobophora variegata*, *Spatoglossum schroederi*, and *Fucus vesiculosus* inhibited HIV reverse transcriptase (RT) [91]. The characterized fucans from Dictyotaceae are heterofucans, containing mainly fucose, galactose, glucose, xylose, and/or uronic acid. The fucan (**72**) from *Fucus vesiculosus* is a homofucan, containing only sulfated fucose. Fucan activity is not only dependent on the ionic changes, but also on the sugar rings that act to spatially orient the charges in a configuration that recognizes the enzyme, thus determining the specificity of the binding [91].

#### 3.8.8. Sulfated Polymannuroguluronate

**Figure 14:**
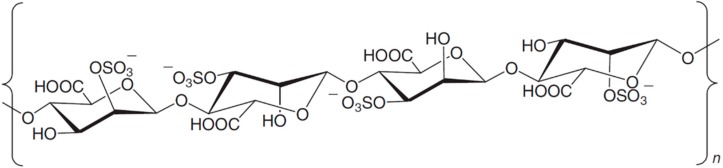
Sulfated polymannuroguluronate (SPMG, **73**) isolated from brown algae *Laminaria japonica* [92].

Sulfated polymannuroguluronate (SPMG, **73**), a new form of sulfated polysaccharide extracted from the brown algae *Laminaria japonica* with an average molecular mass of 8.0 kDa, is rich in 1,4-linked β-d-mannuronate, and has an average of 1.5 sulfates and 1.0 carboxyl groups per sugar residue (Figure 14). SPMG (**73**) has been reported to be in Phase II clinical trials in China as an anti-AIDS drug candidate [92]. SPMG appeared to eliminate the viral gene product known as the transactivator of transcription (Tat) protein, inhibiting its signal transduction and angiogenesis in AIDS-associated Kaposi’s sarcoma cells. In addition, SPMG was noted to block the release of basic fibroblast growth factor (bFGF) and vascular endothelial growth factor (VEGF) from the extracellular matrix (ECM). These results collectively suggest that SPMG functions as a promising therapeutic against Tat-induced angiogenesis and pathologic events relevant to AIDS-Kaposi’s sarcoma. Furthermore, SPMG displays a novel mechanism of anti-AIDS action. Hui *et al.* demonstrated that SPMG appeared to show a neuroprotective effect, because it decreased apoptosis caused by Tat-stimulated calcium overload in PC12 neuronal cells, thus suggesting that SPMG may warrant further clinical studies for HIV-associated dementia [93].

#### 3.8.9. Carraguard

Carraguard (**74**), a sulfated linear polysaccharide of d-galactose and 3,6-anhydro-d-galactose extracted from certain red algae, including *Solieria chordalis* along the coasts of Brittany, has been shown to block HIV and other sexually transmitted diseases *in vitro* [94]. However, phase III clinical trials of Carraguard (**74**), a carrageenan-based compound developed by the Population Council, to determine its efficacy and long-term safety in prevention of HIV infection in women did not detect a significant effect [94].

#### 3.8.10. Aminoethyl-Chitosan, Sulfated Chitin and Sulfated Chitooligosaccharide

Chemical modification of chitin and chitosan generate new biofunctional materials that provide desired biological activities and physicochemical properties [95,96,97].

Aminoethyl-chitosan (**75**), prepared from 50% deacetylated chitosan, showed activity against HIV-1, with an IC_50_ value of 17 μg/mL [98]. Aminoethyl-chitosan (**75**) could be used as a novel drug against HIV. Sulfated chitin (**76**) and chitosan (**77**) have a variety of biological applications, including metal ion adsorption, drug delivery systems, blood compatibility, and antibacterial fields, and anti-HIV-1 effects [95]. Sulfated chitooligosaccharide (SCOS, **78**), which were synthesized by a random sulfation reaction, reportedly possessed anti-HIV activity at low molecular weights (3–5 kDa). At non-toxic concentrations, SCOS (**78**) significantly inhibited HIV-1-induced syncytia formation and lytic effects with EC_50_ values of 2.19 and 1.43 μg/mL, respectively. Furthermore, the production of p24 antigen was suppressed at EC_50_ values of 4.33 and 7.76 μg/mL for HIV-1_RF_ and HIV-1_Ba-L_, respectively. SCOS exhibited inhibitory activities against viral entry and viral-cell fusion by blocking the binding between HIV-1 gp120 and CD4 cell surface receptors. These observations indicated that SCOS might be a novel candidate for the development of anti-HIV-1 agents [99].

### 3.9. Anti-Imflammatory Activity

The inflammatory process can be elicited by numerous internal or external stimuli. Inflammatory diseases are usually treated with drugs that inhibit inflammatory processes. Anti-inflammatory refers to substances or treatments that reduce inflammation. Macrophages are key players in inflammation [100]. Since synthetic anti-inflammatory drugs are known to provoke gastrointestinal irritation, the identification of novel, natural anti-inflammatory drugs and medicines would of great use [101]. Since 2006, two studies reported anti-inflammatory marine carbohydrates.

#### 3.9.1. *Styela plicata* Dermatan Sulfate

**Figure 15:**
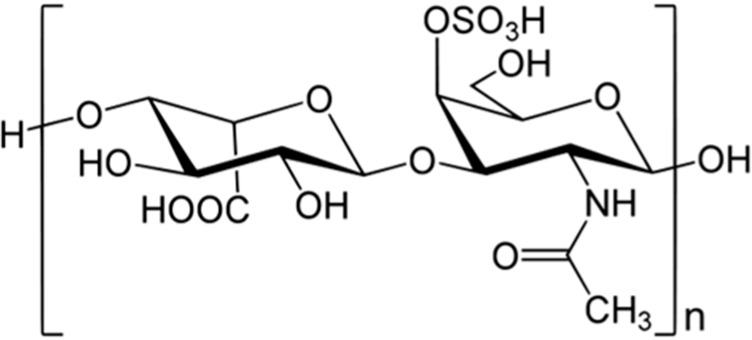
Dermatan sulfate (**79**), isolated from ascidian *Styela plicata* [102].

The anti-inflammatory effect of the mammalian heparin analogue dermatan sulfate (**79**), isolated from the Brazilian ascidian *Styela plicata*, was assessed in a trinitrobenzinesulfonic acid (TNBS)-induced colitis model in rats (Figure 15) [102]. Subcutaneous administration of the dermatan sulfate (**79**) over a 7-day period (8 mg/kg/day) drastically reduced inflammation, as observed by the normalization of the macroscopic and histological characteristics of the colon. At the molecular level, a decrease in the production of tumor necrosis factor α (TNFα), transforming growth factor β (TGFβ), and VEGF was observed, with a concomitant reduction in nuclear factor (NF)-κB and MAPK kinase activation. At the cellular level, the dermatan sulfate (**79**) attenuated lymphocyte and macrophage recruitment, and epithelial cell apoptosis. These results strongly indicate the potential therapeutic use of dermatan sulfate for the treatment of colonic inflammation, with a lower risk of hemorrhage when compared with mammalian heparin [102].

#### 3.9.2. Carijoside A

**Figure 16:**
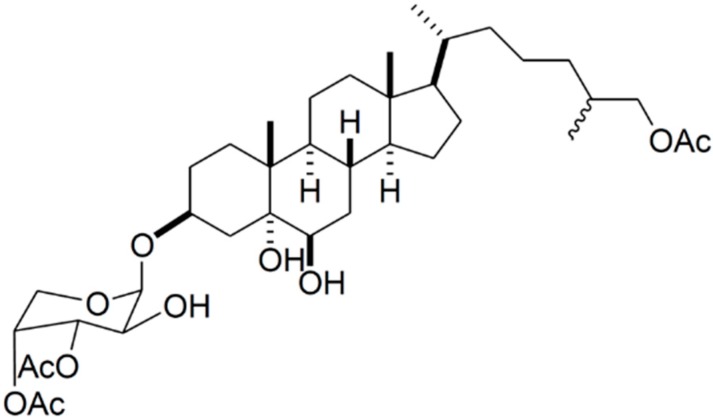
Carijoside A (**80**), isolated from *Carijoa* sp. [103].

A new bioactive sterol glycoside, 3β-*O*-(3',4'-*di*-*O*-acetyl-β-d-arabinopyranosyl)-25ζ-cholestane-3β,5α,6β,26-tetrol-26-acetate (Carijoside A, **80**), was isolated from an octocoral identified as *Carijoa* sp (Figure 16). Carijoside A (**80**) displayed significant inhibitory effects on superoxide anion generation and elastase release by human neutrophils [103]. In an assay of anti-inflammatory activity, carijoside A displayed significant inhibitory effects on superoxide anion generation (IC_50_ = 1.8 μg/mL) and elastase release (IC_50_ = 6.8 μg/mL) by human neutrophils; this compound also exhibited moderate cytotoxicity towards DLD-1 (human colon adenocarcinoma cells), P388D1 (murine macrophage cells), HL-60 (human premyelocytic leukemia), and CCRF-CEM (human T-cell acute lymphoblastic leukemia), with ED_50_ values of 9.7, 10.4, 12.0, and 13.1 μg/mL, respectively [103].

### 3.10. Immunomodulating Activity

Immunomodulation refers to medications that regulate and steer the immunological defense system [104]. Macrophages are the resident immune cells of the innate immune system, and play an important role in the maintenance of homeostasis by changing their function according to the tissue. Furthermore, macrophages are a predominant source of pro-inflammatory factor. It is hypothesized that cancer originates from sites of chronic inflammation, in part based on the hypothesis that some classes of irritants, together with tissue injury and ensuing inflammation they cause, enhance cell proliferation [105]. Some polysaccharides and glycosides obtained from natural sources are considered biological response modifiers and have been shown to enhance various immune responses.

#### 3.10.1. Laminarin Polysaccharides and Oligosaccharides

Laminarin polysaccharides (LP1, **81**) were prepared from *Laminaria japonica*, a marine brown algae with potential biological activity. The molecular weights of the LP1s were between 5 and 10 kDa. Laminarin oligosaccharides (LO, **82**) derived by hydrolyzing LP1 with an endo-β-(1→3)-glucanase from *Bacillus circulans,* were mainly di- and pentaoligosaccharides [106]. Treatment of mouse thymocytes with LO or LP1 (1–4 mg/mL) suppressed apoptotic death around 2- or 3-fold, and extended cell survival in culture by 20%–30%. A mouse cDNA microarray showed that the genes coding for immune response proteins were induced and apoptotic cell death proteins were reduced significantly by LO, providing preliminary information regarding the immunomodulatory mechanism of LO. Laminarin polysaccharides (**81**) and oligosaccharides (**82**) can be utilized to develop new immunopotentiating substances and alternative medicines [106].

#### 3.10.2. ASLP

**Figure 17:**
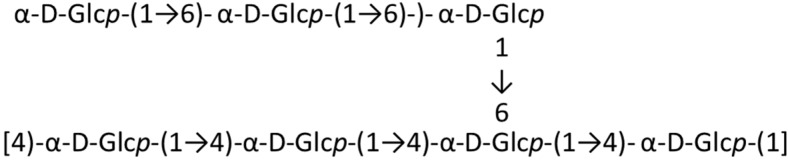
ASLP (**83**) isolated from Lischke *Arca subcrenata* [107].

A water-soluble polysaccharide, ASLP (**83**), was isolated from *Arca subcrenata* Lischke, a popular Chinese seafood [107]. The average molecular weight of ASLP was estimated to be 3500 Da. ASLP is an α-(1→4)-d-glucan, with an α-(1→6)-d-glucan at the C-6 position every fourth residue along the main chain. The branch chain has three glucose residues (Figure 17). ASLP (**83**) stimulated mouse spleen lymphocyte proliferation in a concentration-dependent manner (IC_50_ less than 100 μg/mL). Interestingly, the ASLP branches were required for the immunomodulatory bioactivity [107]. Preliminary immunopharmacological tests suggested that ASLP enhanced spleen lymphocyte proliferation *in vitro*; the branches of ASLP were extremely important for its biological activities.

#### 3.10.3. HCLPS-1

**Figure 18:**
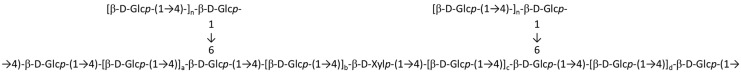
HCLPS-1 (**84**) isolated from clam of *Hyriopsis cumingii* [108].

A water-soluble polysaccharide, HCLPS-1 (**84**), was isolated from the clam *Hyriopsis cumingii* Lea by hot water [108]. HCLPS-1 consists of glucose and xylose in a molar ratio of 35:1, and a main chain of (1→4)-linked β-d-glucopyranosyl residues, with an average molecular weight of 1.56 × 10^5^ Da (Figure 18). HCLPS-1 significantly and dose-dependently promoted concanavalin A (Con A) and lipopolysaccharide (LPS)-stimulated splenocyte proliferation *in vitro.* It also increased Con A- and LPS-induced splenocyte proliferation in mice immunized with sheep red blood cells (SRBC). HCLPS-1 (**84**) could improve both specific and non-specific cellular immune response, and could be explored as a potential natural immunomodulator [108].

#### 3.10.4. Floridoside

Floridoside (**10**), extracted from the red algae *Mastocarpus stellatus*, has a structure similar to the xenoantigen Gal-α-(1→3)-Gal [109]. This xenoantigen induces an enhanced immune response in human xenografts, which are mediated by natural anti-gal antibodies that activate the classical complement pathway. Floridoside (**10**) potently activated the classical complement pathway (IC_50_ = 5.9–9.3 μg/mL) by recruiting immunoglobulin M (IgM), suggesting that the compound might be a novel anti-complementary agent for therapies requiring complement depletion [109].

#### 3.10.5. Frondoside A

**Figure 19:**
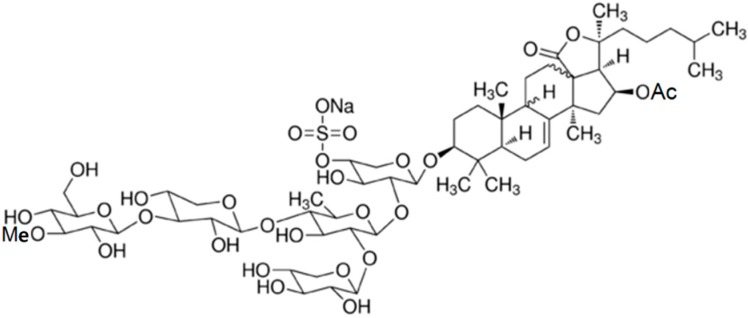
Frondoside A (**85**) isolated from sea cucumber *Cucumaria frondosa* [110].

Frondoside A (**85**), a major triterpene glycoside from the commercially harvested North Atlantic sea cucumber *Cucumaria frondosa*, possesses strong immunomodulatory properties at sub-toxic doses (Figure 19) [110]. Frondoside A was shown to stimulate lysosomal activity and phagocytosis in mouse macrophages, as well as ROS formation. Frondoside A has a weak effect upon IgM production after immunization with sheep erythrocytes in mice. Frondoside A does not stimulate Ig production in mice, and does not significantly enhance ovalbumin-stimulated IgM and IgG antibody levels. Frondoside A stimulates cell-based immunity, including phagocytosis, without a significant effect on humoral immune activity or adjuvant properties. In addition, frondoside A showed anticancer activities *in vitro* and suppressed tumor growth *in vivo* [111]. The antitumor activity of frondoside A (**85**) resulted from its induction of apoptosis in cancer cells [111,112]. The cancer inhibitory effect of frondoside A in tumor-bearing mice might also partly result from its other biological activities, including antiangiogenic and antimetastatic effects [111,113,114]. Holt *et al.* investigated the effect of frondoside A on NK cells and demonstrated that prostaglandin E2 (PGE2) significantly suppressed the secretion of interferon-γ (IFNγ) in these cells [115]. Therefore, frondoside A (**85**) may provide curative and/or preventive treatment options against immune diseases.

#### 3.10.6. Cucumariosides

**Figure 20:**
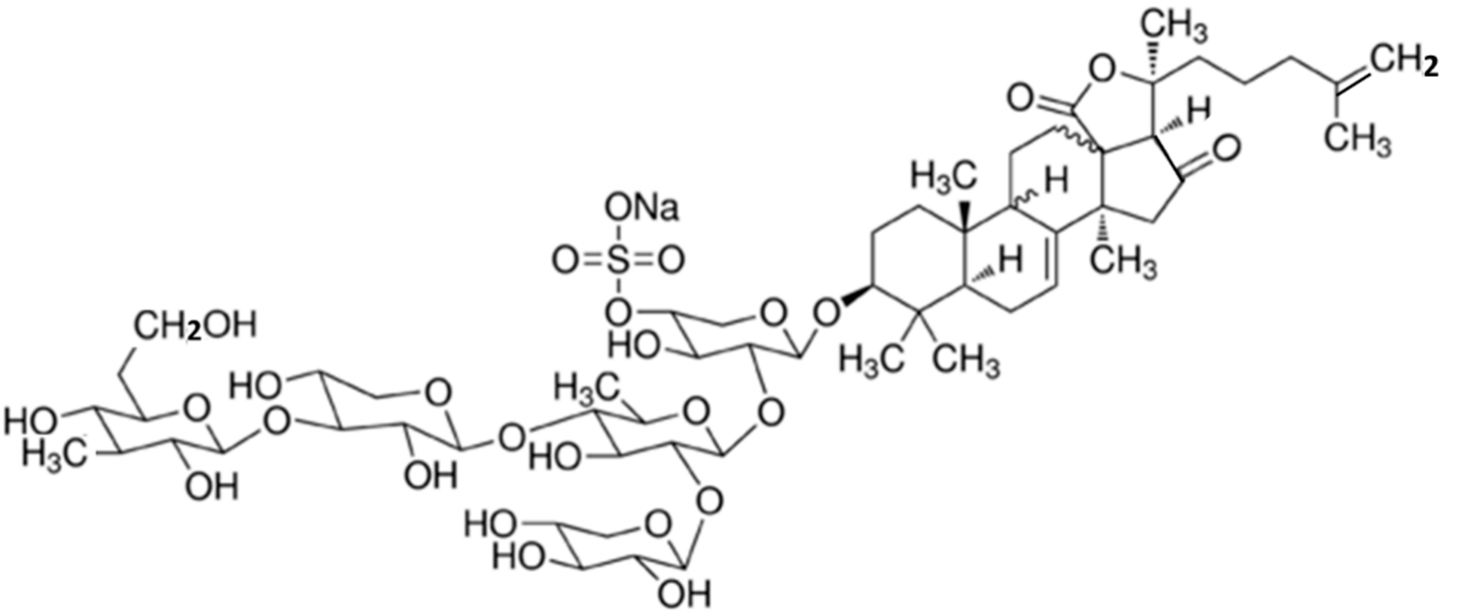
Cucumariosides (especially most active glycoside cucumarioside A2-2, **86**) isolated from sea cucumber *Cucumaria japonica* [116].

Aminin *et al.* investigated the immunomodulatory properties of a “medical lead” called cumaside, which consists of cholesterol complexed with monosulfated cucumariosides (especially most active glycoside cucumarioside A2-2, **86**) and triterpene oligoglycosides from the Far-Eastern edible sea cucumber *Cucumaria japonica* (Figure 20) [116]*.* The hemolytic activity of cumaside was significantly reduced in comparison with that of the original glycosides, due to the glycoside-cholesterol complex. Exposure of mouse macrophages to low concentrations of cumaside produced more than two-fold stimulation of lysosomal activity. This preparation was found to significantly increase animal resistance to bacterial infections elicited by various pathogens. It stimulated phagocytosis, ROS formation, IL6, and TNF-α production in lymphocytes, increased the number of antibody-producing cells, and amplified the expression of several cell surface molecules (CD3, CD4, CD8) in response to hydrocortisone. However, this preparation did not affect the delayed-type hypersensitivity, proliferative activity of lymphocytes, the cytotoxic activity of NK-cells, or cytokine IFNγ and IL12p70 release [116]. The investigators observed that cumaside, while lowering the membranolytic activity of the cucumariosides, appeared to significantly enhance their immunomodulatory properties in both human and murine macrophages and lymphocytes.

However, cucumariosides increased the lysosomal activity and intracellular Ca^2+^ concentrations of macrophages. These effects were related to the chemical structures of the molecules. For example, although there was no direct correlation, Silchenko *et al.* suggested that the lysosomal activity and cytotoxicity of cucumariosides depended on features of both the aglycone and the carbohydrate chain [117]. Other studies reported that *in vitro* treatment of peritoneal macrophages with cucumarioside A2-2 (**86**) stimulated cell adhesion, as well as their spreading reaction and motility [118]. Therefore, it is important to compare the effects of cucumariosides on the migration and spreading of various kinds of cells, including cancer and immune cells.

Silchenko *et al.* [119] isolated three new minor triterpene glycosides, cucumariosides I1 (**87**), I3 (**88**), and I4 (**89**). These have branched pentasaccharide carbohydrate moieties with two sulfate groups and possess 3-*O*-methyl-d-xylose as a terminal monosaccharide unit; this is a characteristic feature of all glycosides isolated from *E. fraudatrix*. Cucumariosides I1 (**87**) and I3 (**88**) differ from each other by the side chain structures in the holostane aglycone moieties, while cucumarioside I4 (**89**) has a 23,24,25,26,27-pentanorlanostane aglycone, with an 18 (**16**)-lactone [119]. The cytotoxic activities of cucumariosides I1, I3, and I4 against mouse spleen lymphocytes and the ascites from mouse Ehrlich carcinoma cells were studied, along with their hemolytic activity in mouse erythrocytes and their antifungal activity. Cucumarioside I1 has an aglycone side chain with a 24 (**25**)-double bond and possessed moderate activity in all of these tests. In contrast, cucumarioside I3, with a 23 (**24**)-double bond and a 25-hydroxy group in the side chain, and cucumarioside I4, containing an aglycone with an 18 (**16**)-lactone and a shortened side chain, either showed low activities or were inactive [119].

## 4. Conclusions

In this review, we discuss the recently identified carbohydrates and glycosides of marine origin, and their medical and pharmaceutical applications. Many experimental results have revealed novel, exciting, and promising marine sources of carbohydrates and glycosides for medical applications. Marine oligosaccharides possess various biological activities that could be useful in drugs. However, although numerous bioactivity marine carbohydrate and glycosides have been identified, their detailed molecular mechanisms remain unknown. It is hard to predict how exactly these molecules exert their activity. Therefore, future research should be directed towards understanding the molecular mechanisms of these compounds. Further investigations with a multidisciplinary approach are imperative to develop novel carbohydrates and glycosides for novel therapeutics.

**Table 1:** List of bioactive carbohydrates and glycosides from diverse marine sources.

Activity	Compound/Chemistry	Source of Original Carbohydrate	Pharmacologic Activity	Inhibitory Concentrations	Refs.
Antioxidant	HCPS-3 (**1**)/polysaccharides	Pearl mussel: *Hyriopsis cumingii*	Inhibition of MDA formation *in vivo*	ND	[34]
Antioxidant	c-EPL (**2**)/polysaccharide	Fungus: *Cerrena unicolor*	Scavenging activity against DPPH^−^ and ABTS	ND	[35]
Antioxidant	MP-I (**3**)/polysaccharide	Mussel: *Mytilus coruscus*	ALT, AST, and MDA inhibition *in vivo*	ND	[36]
Antioxidant	HWS (**4**)/polysaccharide	Mussel: *Mytilus coruscus*	Scavenging activity against O^−^, OH^−^, and NO_2_^−^	ND	[37]
Antioxidant	PS1-1 (**5**), PS1-2 (**6**), PS2-1 (**7**)/polysaccharide	Fungus: *Penicillium* sp. *F23-2*	Scavenging activity against DPPH^−^, O^−^, and OH^−^	2.53–6.81 mg/mL (EC_50_)	[38]
Antioxidant	*S. swartzii* sulfate polysaccharide (**8**)	Algae: *Sargassum swartzii*	Scavenging activity against ABTS, H_2_O_2_ and DPPH^−^	ND	[39]
Antioxidant	NA-COS (**9**)/oligosaccharide	Crab: *Chionoecetes opilio*	Scavenging activity against DPPH^−^, hydroxyl radical, and alkyl radical	0.8–1.75 mg/mL (IC_50_)	[41]
Antioxidant	Floridoside (**10**), d-isofloridoside (**11**)/glycoside	Algae: *Laurencia undulate*	Inhibition of free-radical oxidative stress	22–43 μM (IC_50_)	[42]
Antidiabetic	Fucoidan (**12**)/polysaccharide	Algae: *Fucus vesiculosus*, *Ecklonia kurome*, *Undaria pinnatifida*	Decrease blood glucose, total cholesterol, and fat adiponectin levels *in vivo*	ND	[45,46,47,48,49]
Antidiabetic	Aquastatin A (**13**)/glycoside	Fungus: *Cosmospora* sp. SF-5060	Tyrosine phosphataseinhibition	0.19 μM (IC_50_)	[50]
Antibacterial	*S. pharaobis* polysaccharide (**14**)	Cuttlebone: *Sepia pharaonis*	*Staphylococcus aureus*, *Escherichia coli*, *Salmonella typhii*, *Vibrio cholerae*, *Klebsiella oxytoca*, *Salmonella paratyphi*, *Proteus mirabilis*, and *Staphylococcus pyogenes* inhibition	40–100 mg/mL (MIC)	[52]
Antibacterial	Gladius polysaccharide (**15**)	Cuddalore: *Loligo duvauceli*, Mudasalodai: *Doryteuthis sibogae*	*Bacillus subtilis*, *Shigella* sp., *S. typhii*, *Vibrio parahaemolyticus*, *Klebsiella pneumonia*, and *E. coli* inhibition	80–100 mg/mL (MIC)	[53]
Antibacterial	Caminoside A–D (**16**–**19**)/glycoside	Sponge: *Chaetomorpha sphaeoroconia*	Methicillin-resistant *S. aureus*, vancomycin-resistant *Enterococcus*, and *E. coli* inhibition	6.3–12.5 μg/mL (MIC)	[54,55,56]
Antibacterial	*C. sphaeoroconia* sulfate galactan (**20**)/polysaccharide	Algae : *Caminus sphaeoroconia*	*B. subtilis, Micrococus lutens* and *S.aureus* inhibition	40 mg/mL (MIC, MBC: *S. aureus*)	[57]
Antifungal	Triterpene glycoside (**21**), holothurin B (**22**), holothurin A (**23**)/glycoside	Sea cucumber: *Actinopyga lecanora*	*Candida albicans*, *Cryptococcus neoformans*, *Sporothrix schenckii*, *Trychophyton mentagrophytes*, and *Aspergillus fumigatus* inhibition	1.56–50 μg/mL (MIC)	[60]
Antifungal	Marmoratoside A (**24**), 17α-hydroxy impatienside (**25**), marmoratoside B (**26**), 25-acetoxy bivittoside (**27**)/glycoside	Sea cucumber: *Bohadschia marmorata*	*C. albicans*, *C. neoformans*, *A. fumigatus*, *Trichophyton rubrum*, *Candida tropicalis*, and *Candida krusei* inhibition	1–16 μg/mL (MIC_80_)	[61]
Antifungal	Scabraside A (**28**), ethinodea A (**29**), holothurin A1 (**30**)/glycoside	Sea cucumber: *Holothuria scabra*	*C. albicans*, *C. neoformans*, *Candida pseudotropicalis*, *T. rubrum*, *Fonsecaea compacta*, *A. fumigatus*, and *Microsporum gypseum* inhibition	1–16 μg/mL (MIC_80_)	[62]
Antifungal	Sokodoside A (**31**), B (**32**)/glycoside	Sponge: *Erylus placenta*	*Mortierella ramanniana*, *Saccharomyces cerevisiae* inhibition	ND	[64]
Antifungal	Variegatuside C–F (**33**–**36**)/glycoside	Cucumber: *Stichopus variegates*	*C. albicans*, *C. neoformans*, *C. pseudotropicalis*, *C. parapsilosis*, *C. tropicalis and Microsporum gypseum* inhibition	3.4–12.5 μg/mL (MIC_80_)	[65]
Anticoagulant	Fucoidan (**12**)/polysaccharide	Algae: *Fucus vesiculosus*, *Ecklonia kurome*, *Undaria pinnatifida*	Thrombin and factor Xa inhibition *in vitro* and *in vivo*	ND	[69,70]
Anticoagulant	*L. catenata* acid polysaccharide (**37**)	Algae: *Lomentaria catenata*	Thrombin and factor Xa inhibition *in vitro*	0.045–25.47 USP units/mg	[71]
Anticoagulant	Brown algae Sulfate polysaccharide (**38**–**42**)	Algae : *Sargassum tenerrimum* (**38**), *S. wightii* (**39**), *Turbinaria conoides* (**40**), *T. ornata* (**41**), *Padina tetrastromatica* (**42**)	Thrombin inhibition and heparinoid activity *in vitro*	14.5–25.41 haparin USP units/mg	[72]
Anticoagulant	AMP-2 (**43**)/polysaccharide	Cucumber: *Acaudina molpadioidea*	Thrombin, prothrombin and thrombin inhibition *in vitro*	11.8–34.3 at 0.5 mg/mL	[73]
Anticoagulant	*L. catenata* polysaccharide (**44**)	Algae: *Lomentaria catenata*	Thrombin and factor Xa inhibition *in vitro*	183 IU/mg	[74]
Anticoagulant	Hf-SP/polysaccharide (**45**)	Seaweed: *Halymenia floresia*	Thrombin inhibition *in vitro*	10.72 IU/mg	[75]
Anticoagulant	*A. taxiformis* sulfated polysaccharide (**46**)	Algae: *Asparagopsis taxiformis*	Thrombin inhibition *in vitro*	259.8 μg/mL	[76]
Anticoagulant	*C. cupressoides* polysaccharide 2 (**47**)	Algae: *Caulerpa cupressoides var. lycopodium*	Thrombin and factor Xa inhibition *in vitro*	ND	[77]
Antiprotozoal	Pandaroside G (**48**), pandaroside G methyl ester (**49**)/glycoside	Sponge: *Pandaros acanthifolium*	*Trypanosoma brucei rhodesiense* and *Leishmania donovani* inhibition	0.038–1.3 μM (IC_50_)	[78]
Antiprotozoal	Acanthifoliosides A–F (**50**–**55**)/glycoside	Sponge: *Pandaros acanthifolium*	*T. brucei rhodesiense* and *L. donovani* inhibition	5.7–94.8 μM (IC_50_)	[79]
Antimalarial	*M. austere* glycosides (**56**-**63**)	Octocoral: *Muricea austere*	*Plasmodium falciparum* inhibition	21–80 μM (IC_50_)	[82]
Antiviral	Naviculan (**64**)/polysaccharide	Diatom: *Navicula directa*	HSV-1 and HSV-2 inhibition	7–14 μM (IC_50_)	[85]
Antiviral	PSC (**65**), PBT (**66**)/polysaccharide	Algae: *Sphaerococcus coronopifolius* (PSC), *Boergeseniella thuyoides* (PBT)	HSV-1 inhibition	4.1–17.2 μg/mL (EC_50_)	[86]
Antiviral	Sulfate GFP (**67**), GLPE (**68**)/polysaccharide	Algae: *Grateloupia longifolia*	HIV-1 inhibition	0.003–0.010 μg/mL (EC_50_)	[87]
Antiviral	d,l-galatan hybrid C2S-3 (**69**)/polysaccharide	Seaweed: *Cryptonemia crenulata*	Dengue type 2 inhibition	0.8–16 μg/mL (IC_50_)	[88]
Antiviral	Sulfate xylomannan (**70**)/polysaccharide	Seaweed: *Scinaia hatei*	HSV-1 and HSV-2 inhibition	0.5–1.4 μg/mL (IC_50_)	[89]
Antiviral	Galactofucan EA-20 (**71**)/polysaccharide	Algae: *Adenocystis utricularis*	HIV-1 inhibition	0.6 μg/mL (IC_50_)	[90]
Antiviral	Sulfate fucans (**72**)/polysaccharide	Seaweed: *Dictyota mertensii*, *Lobophora variegata*, *Spatoglossum schroederi*, *Fucus vesiculosus*	HIV-1 reverse transcriptase inhibition	ND	[91]
Antiviral	Sulfate SPMG (73)/polysaccharide	Algae: *Laminaria japonica*	Inhibition of HIV-1 infection	ND	[92,93]
Antiviral	Carraguard (**74**)/polysaccharide	Algae: *Solieria chordalis*	HIV-1 inhibition	ND	[94]
Antiviral	Aminoethyl-chitosan (**75**), sulfated chitin (**76**), chitosan (**77**)/polysaccharide	Fungus: *Zygomycetes*, Alage: *Chlorella* sp. Crab, crayfish, periwinkle and shrimp	HIV-1 inhibition	17 μg/mL (IC_50_)	[95,98]
Antiviral	Sulfated SCOS (**78**)/oligosaccharide	Fungus: *Zygomycetes*, Alage: *Chlorella* sp. Crab, crayfish, periwinkle and shrimp	HIV-1 inhibition	1.4–7.76 μg/mL (IC_50_)	[99]
Anti-inflammatory	Dermatan sulfate (**79**)/polysaccharide	Ascidian: *Styela plicata*	Colonic inflammation inhibition	8 mg/kg (IC_50_)	[102]
Anti-inflammatory	Carijoside A (**80**)/glycoside	Coral: *Carijoa* sp.	Neutrophil superoxide and elastase inhibition	1.8–6.8 μg/mL	[103]
Immune system	Laminarin polysaccharide LP1 (**81**), Laminarin oligosaccharide LO (**82**)	Alage: *Laminaria japonica*	Inhibition of lymphocyte apoptosis	1–4 mg/mL	[106]
Immune system	ASLP (**83**)/polysaccharide	Lischke: *Arca subcrenata*	Increases splenocyte proliferation	<100 μg/mL (IC_50_)	[107]
Immune system	HCLPS-1 (**84**)/polysaccharide	Clam: *Hyriopsis cumingii*	*In vivo* & *in vitro* T and B cell activation	20 mg/kg (IC_50_)	[108]
Immune system	Floridoside (**10**)/glycoside	Algae: *Mastocarpus stellatus*	Stimulation of IgM	5.9–9.3 μg/mL (IC_50_)	[109]
Immune system	Frodoside A (**85**)/glycoside	Sea cucumber: *Cucumaria frondosa*	Lysosomal activity, phagocytosis and ROS activation	0.1–0.001 μg/mL	[110]
Immune system	Cucumarioside A2-2 (**86**), I1 (**87**), I3 (**88**), I4 (**89**)/glycoside	Sea cucumber: *Cucumaria japonica*, *Eupentacta fraudatrix*	Simulation of lymphocytes and neutrophils	ND	[116,117,118,119]

ND: not determined; MDA: malondialdehyde; DPPH: 2,2-diphenyl-1-picrylhydrazyl; ABTS: 2,2'-azino-bis(3-ethylbenzothiazoline-6-sulphonic acid); ALT: alanine aminotransferase; AST: aspartate aminotransferase; HIV: human immunodeficiency virus; HSV: herpes simplex virus; ROS: reactive oxygen species.
